# Accelerated aging in normal breast tissue of women with breast cancer

**DOI:** 10.1186/s13058-021-01434-7

**Published:** 2021-05-22

**Authors:** Shoghag Panjarian, Jozef Madzo, Kelsey Keith, Carolyn M. Slater, Carmen Sapienza, Jaroslav Jelinek, Jean-Pierre J. Issa

**Affiliations:** 1 Coriell Institute for Medical Research, 403 Haddon Ave., Camden, NJ 08103 USA; 2 Fox Chase Cancer Center, Philadelphia, PA USA; 3 Fels Institute for Cancer Research, Temple University School of Medicine, Philadelphia, PA USA

**Keywords:** DNA methylation, Outliers, Accelerated aging, Predicted age

## Abstract

**Background:**

DNA methylation alterations have similar patterns in normal aging tissue and in cancer. In this study, we investigated breast tissue-specific age-related DNA methylation alterations and used those methylation sites to identify individuals with outlier phenotypes. Outlier phenotype is identified by unsupervised anomaly detection algorithms and is defined by individuals who have normal tissue age-dependent DNA methylation levels that vary dramatically from the population mean.

**Methods:**

We generated whole-genome DNA methylation profiles (GSE160233) on purified epithelial cells and used publicly available Infinium HumanMethylation 450K array datasets (TCGA, GSE88883, GSE69914, GSE101961, and GSE74214) for discovery and validation.

**Results:**

We found that hypermethylation in normal breast tissue is the best predictor of hypermethylation in cancer. Using unsupervised anomaly detection approaches, we found that about 10% of the individuals (39/427) were outliers for DNA methylation from 6 DNA methylation datasets. We also found that there were significantly more outlier samples in normal-adjacent to cancer (24/139, 17.3%) than in normal samples (15/228, 5.2%). Additionally, we found significant differences between the predicted ages based on DNA methylation and the chronological ages among outliers and not-outliers. Additionally, we found that accelerated outliers (older predicted age) were more frequent in normal-adjacent to cancer (14/17, 82%) compared to normal samples from individuals without cancer (3/17, 18%). Furthermore, in matched samples, we found that the epigenome of the outliers in the pre-malignant tissue was as severely altered as in cancer.

**Conclusions:**

A subset of patients with breast cancer has severely altered epigenomes which are characterized by accelerated aging in their normal-appearing tissue. In the future, these DNA methylation sites should be studied further such as in cell-free DNA to determine their potential use as biomarkers for early detection of malignant transformation and preventive intervention in breast cancer.

**Supplementary Information:**

The online version contains supplementary material available at 10.1186/s13058-021-01434-7.

## Background

Methylation of human DNA comprises the biochemical addition of a methyl (CH_3_) group, primarily on a cytosine when followed by a guanosine (CpG). DNA methylation, once established, is a stable signal which serves as a regulatory mechanism for gene expression as well as a memory signal [1–3]. However, it is now well established that DNA methylation alters with age in normal healthy individuals and in disease states. In early studies that focused on a handful of genes, it was reported that DNA methylation increased linearly with age [4, 5]. Later, the advances in whole-genome quantitative analysis of DNA methylation enabled the identification of specific loci with gains and losses of methylation. In fact, we reported that this epigenetic drift is correlated with lifespan and is conserved across species [6, 7]. It was also reported that DNA methylation across different tissues could be used as a biomarker to predict the biological (epigenetic) age [8–10]. Of interest are individuals with accelerated epigenetic aging, who have acquired altered methylation faster than expected based on their chronological age. Exploring these extreme outlying variations in DNA methylation in normal tissues could help explain biological variations in disease states. However, these extreme DNA methylation alterations in normal tissues are infrequent events, making these stochastic outlier events that are difficult to identify. Several recent studies reported on different algorithms used to identify these rare events [11–13].

On the other hand, alteration in DNA methylation, such as global hypomethylation and localized hypermethylation at gene promoters, is a hallmark of cancer [14]. In breast cancer, aberrant DNA methylation signatures are closely associated with the different molecular subtypes [15, 16]. Additionally, tumor suppressor genes that gain DNA methylation at their promoters may be inactivated in breast cancer, with reports on over 100 candidate genes with promoter hypermethylation that potentially play significant roles in driving the disease [17–19]. Interestingly, many of the DNA methylation changes reported in cancer are also observed in normal aging tissues, such as the breast tissue, and age-dependent hypermethylated genes are frequently hypermethylated in cancer [11, 20–22]. The comparison of the alterations of DNA methylation between normal and cancer tissue is important in defining a potential field defect.

However, despite the efforts to identify individuals with extreme epigenetic age variations (outliers), it is still unclear what roles age-dependent DNA methylation outliers play in normal breast and in breast cancer. Therefore, the goal of this study was to detect tissue-specific age-dependent DNA methylation changes in normal breast tissue and identify individuals with methylation outliers and accelerated epigenetic age. Following from that, this study concludes by exploring what role age-related DNA methylation outliers play in epigenetic field defects and in carcinogenesis.

## Methods

### Purification of normal breast epithelia

Twenty-nine human mammary epithelial cell (HMEC) lines were utilized as starting material for the identification of age-dependent DNA methylation sites. Under an approved protocol by the Institutional Review Board (IRB) at Fox Chase Cancer Center, the primary human mammary epithelial cells were routinely derived from adjacent or contralateral normal mammary tissue of breast cancer patients using an established commercial protocol of EpiCult®-B human mammary epithelial cell culture (Stemcell Technologies, BC, Canada) as previously described [23]. Established primary HMEC lines were maintained in culture for 4 passages in medium containing 1:1 DMEM/F12 (Life Technologies, Carlsbad, CA), 2.438 g/L sodium bicarbonate, 5% chelated horse serum, 20 ng/mL EGF (BD Biosciences, San Jose, CA), 100 ng/mL cholera toxin (Sigma-Aldrich, St. Louis, MO), 10 mg/L insulin (SigmaAldrich, St. Louis, MO), 0.5 mg/L hydrocortisone (Sigma-Aldrich, St. Louis, MO), antibiotic-antimycotic (Life Technologies, Carlsbad, CA), and 0.04 mM calcium chloride (Sigma-Aldrich, St. Louis, MO). Genomic DNA was isolated from HMEC lines by phenol-chloroform extraction as previously described [24]. The normal adjacent tissue samples were collected > 2 cm from the tumor margin, and the H&E slides were reviewed and scored by independent pathologists [15, 25].

### DNA methylation profiling

To analyze the genome-wide DNA methylation profile, we used Digital Restriction Enzyme Analysis of Methylation (DREAM) as described previously [26]. Briefly, DREAM is a quantitative mapping of DNA methylation with high resolution on a genome-wide scale. The method is based on sequential cuts of genomic DNA with a pair of neoschizomer endonucleases (*SmaI* and *XmaI*) recognizing the same restriction site (CCCGGG) containing a CpG dinucleotide (CG). *SmaI* cuts first all unmethylated sites at CCC^GGG while the methylation tolerant *XmaI* follows by cutting the methylated sites at C^CCGGG. The enzymes thus generate distinct methylation-specific signatures at the ends of DNA fragments which are deciphered by next-generation sequencing. The methylation level at individual CpG sites is calculated as the ratio of sequencing reads with the methylated signature CCGGG to the total number of reads mapping to the site. Using the DREAM method, we analyzed the methylation profiles of the normal adjacent human mammary epithelial cells (*n* = 29). Pair-end sequencing of 40 bases was performed on HiSeq 2500 (Illumina, San Diego, CA, USA) instrument at the Genomic Core Facility of Fox Chase Cancer Center (Philadelphia, PA, USA). These sequence data have been submitted to the GEO database under accession number GSE160233. We mapped the sequences to the human genome (hg19) and calculated the methylation at target sites. Unsupervised hierarchical clustering (clustering = ward.D2, distance = Euclidean) and heatmap were generated in R using the pheatmap library.

### DNA methylation datasets

Publicly available DNA methylation datasets (Illumina HumanMethylation 450K array) from normal breast tissue (GSE88883, GSE74214, GSE101961) and from normal adjacent to cancer breast tissue (TCGA, Firehose Legacy) were re-normalized within themselves to match the normalization of the GSE69914 dataset for which raw array files were not available for normalization. The ChAMP R package was used for normalization, first filtering out low-quality probes, then imputing the missing values with champ.filter(), followed by re-normalizing with champ.norm() using the default method beta-mixture quantile normalization (BMIQ) [27, 28]. All datasets were used to identify outliers of DNA methylation.

### Identification and validation of age-dependent sites

For our discovery dataset, to identify CpG sites with methylation changes due to age, we generated DNA methylation sequencing data for 29 of the purified normal adjacent human breast epithelia (age range 33–82 years old) using DREAM (GSE160233). To validate the age-related sites identified based on permutation analysis of the DREAM dataset, we used DNA methylation (450K array) of 97 normal adjacent TCGA samples. The details of the discovery and validation of the age-related sites are further explained in the “Results” section.

### Identification of outlier samples and age prediction

To detect DNA methylation outlier samples, we first ran principal component analysis (PCA) on the validated 146 age-related sites (described in “Results” section) across 427 patient samples from the DNA methylation datasets mentioned above. Next, we calculated an unsupervised anomaly detection parameter, the local outlier factor (LOF) on all the principal components of PCA using the DMwR and Rlof packages in R [29, 30]. The LOF algorithm computes an outlier score based on the local density deviation of a given data point with respect to the neighboring points. LOF uses a parameter *k* (the number of neighboring points) to calculate the local reachability density (lrd) which is the optimal distance from the neighbor to the individual data point. LOF is then calculated based on the average ratio of local reachability densities of the neighboring points to the local reachability density of the data point according to the following equation:

$$ {\mathrm{LOF}}_k(o)=\frac{\sum_{o^{\prime}\epsilon {N}_k(o)}\frac{{\mathrm{lrd}}_k\left({o}^{\prime}\right)}{{\mathrm{lrd}}_k(o)}}{\left|\left|{N}_k(o)\right|\right|}=\sum \limits_{o^{\prime}\epsilon {N}_k(o)}{\mathrm{lrd}}_k\left({o}^{\prime}\right).\sum \limits_{o^{\prime}\in {N}_k(o)}{\mathrm{reachdist}}_k\left({o}^{\prime}\leftarrow o\right). $$

In the above formula, *o* is the object, *N*_*k*_(*o*) is the set of the *k* nearest neighbors, *o′* is the neighboring object used in calculating the reachability distance of *o* from *o′* but at least *k*-distance of *o′*. If the density of a point is much smaller than the densities of its neighbors, then the point is considered an outlier. To statistically determine the cutoff for the outlier scores calculated by the LOF algorithm, we calculated the interquartile range (IQR) for the outlier scores, and samples with an outlier score of ≥ Q3 +1.5 × IQR were designated as outlier samples. We used the least absolute shrinkage and selection operator (Lasso) to regress the age of the samples based on the DNA methylation. The glmnet R [31] package was used to implement the Lasso model with the penalty parameter fitted by cross-validation. The cv.glmnet function parameters were set as follows: family = “gaussian,” type.measure = “mse,” alpha = 1, and the remaining parameters were set by default. For the age prediction, we used a model with the largest value of lambda such that the error is within 1 standard error of the minimum (lambda.1se) determined by cross-validation model fitting.

### Statistical analysis

Ten thousand or 1000 random permutations of the age of the patients, and the DNA methylation samples were used to statistically analyze age-related DNA methylation changes. The age-dependent methylation changes were selected based on the cutoffs of permutation empirical *p* value (*p*<0.05) and Spearman correlation of *r* ≥ 0.3 and *r* ≤ −0.3. Unsupervised hierarchical clustering was performed in R using Ward’s method implemented in the hclust function. Quantitative DNA methylation differences were defined as a difference in the average beta value across conditions greater than 0.2 and an FDR < 0.001. A chi-square test was used to test the significance for each odds ratio comparison and *p* values indicated. All publicly available DNA methylation datasets were renormalized using the beta-mixture quantile normalization through the ChAMP R package. Outlier scores were calculated by LOF using DMwR and Rlof packages in R. The Lasso regression model was built using the glmnet R package for age prediction with the penalty parameter fitted by cross-validation. The significance of the differences in the predicted and chronological ages was tested by the Wilcoxon test and in paired patient samples across different tissue types by the Kruskal-Wallis test followed by a post hoc Dunn test with multiple testing correction using the Benjamini-Hochberg method. The significance of the overlap of outlier patients was tested by the hypergeometric test. The significance of the mutation levels was calculated using the Fisher exact test. The significance of the clinical characteristics for the three different groups was calculated using ANOVA testing followed by Tukey’s HSD post hoc test.

## Results

### Identification and characterization of age-dependent sites with DNA methylation changes in normal breast epithelium

**Fig. 1:**
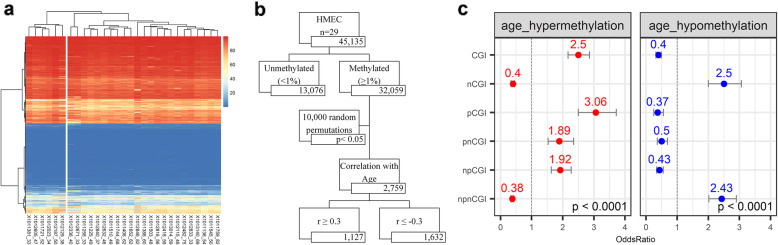
DNA methylation changes in normal breast epithelium. **a** Unsupervised hierarchical clustering of 29 purified human mammary epithelial (HMEC) samples based on 45,135 CpG sites with more than 100 sequencing reads in at least 75% of the samples. DNA methylation levels are shown as the blue-red gradient. **b** Flowchart of steps taken to find CpG sites associated with age. Starting with all CpG sites detected, sites with greater than 1% methylation were used for a permutation test using the Spearman correlation between DNA methylation and age. Ten thousand random permutations of the age of the patient samples were used to statistically analyze age-related DNA methylation sites. The age-dependent methylation changes were selected based on a cutoff of permutation empirical *p* value of (*p* < 0.05) and based on a Spearman correlation of *r* ≥ 0.3 (gain of methylation with age) and r ≤ −0.3 (loss of methylation with age). The numbers in each box indicate the number of DNA methylation sites. **c** Summary of odds ratios of the genomic region specificity of age-related hypermethylated (red) and hypomethylated (blue) sites compared to non-age-related sites. Red and blue dots represent the point estimates of the odds ratio for hypermethylation and hypomethylation, respectively, with lines representing the 95% confidence intervals around the estimates. A chi-square test was used to test for statistical significance for each comparison; *p* values for all comparisons were lower than 0.0001. Genomic regions are defined as follows: CGI (CpG island), nCGI (non-CpG island), pCGI (promoter CpG island), npCGI (non-promoter CpG island), pnCGI (promoter non-CpG island), and npnCGI (non-promoter non-CpG island)

Owing to the evidence that normal breast tissue is comprised largely of adipose cells [32], we sought to identify and characterize age-dependent DNA methylation changes in purified human breast epithelium from which cancer arises. We generated DNA methylation sequencing data for 29 of the purified normal adjacent human breast epithelia (age range 33–82 years old) using DREAM (GSE160233). We chose to use DREAM methodology in our discovery analysis because it is robust, highly reproducible, and has a background of less than 1%, making it ideal for the accurate detection of low methylation levels and small changes such as the ones observed in aging [26]. To identify the sites with methylation changes due to age, we first examined the clustering of the samples based on all sites (45,135) with more than 100 reads in 75% of the samples. The unsupervised hierarchical clustering (Fig. 1a) of these sites divided the samples into two groups: first, a cluster of 6 patients with an average age of 42 and, second, a cluster of 23 patients with an average age of 55. The difference in the average ages of the two clusters was significant by the unpaired *t* test (*p* value = 0.01). To identify the sites with methylation changes due to age, we calculated the Spearman correlation between the methylation of CpG sites with more than 1% average methylation (32,059 sites) in these 29 breast epithelia and the age of the patients. To statistically analyze age-related DNA methylation changes, 10,000 random permutations were performed on the ages of the patient samples with the methylation data, and empirical *p* values were computed. We selected 2759 age-related sites based on a cutoff of *r* ≥ 0.3 (for gain of methylation) and *r* ≤ −0.3 (for loss of methylation) with empirical *p* value < 0.05 (Fig. 1b). Next, we characterized the 2759 aging sites which represented 8.6% of the dataset (32,059 sites). Forty-one percent (1127/2759) of the aging sites gained DNA methylation with age while 59% (1632/2759) of the aging sites lost methylation with age. Furthermore, we showed that 304 of the 1127 age-related sites that gained DNA methylation were enriched at CpG islands (CGI), particularly at the promoter regions (pCGI, OR 3.06, 95% CI 2.52–3.72), compared to sites that did not show age-related changes (3777 CGI sites out of 29,299 sites) (Fig. 1c in red). On the other hand, non-CGI regions, particularly non-promoter non-CGI regions (npnCGI, OR 2.43, 95% CI 2.52–2.9), were more likely to lose methylation with age compared to non-age-related sites (24,137 out of 29,299 sites) (Fig. 1c in blue).

### Validation of age-related sites

To validate the age-related sites identified based on the permutation analysis of the DREAM dataset (*n*=29), we used DNA methylation (450K array) of 97 normal adjacent TCGA samples (Additional file 1: Figure S1b). Considering the high background in this platform, we excluded sites with less than 10% methylation. To analyze the correlation between age and methylation, we performed 1000 random permutations of the Spearman correlation between DNA methylation and age and computed the empirical *p* values to measure the significance. The distribution of the Spearman correlation *r* values in the actual dataset (Additional file 1: Figure S1a, pink) showed a marked excess of positive correlation values (gain of methylation) and some negative correlation values (loss of methylation) compared to the distribution of the correlation values obtained by random permutation analysis of the same data (Additional file 1: Figure S1a, green). We used the same cutoff as in our discovery set (0.3 ≤ *r* ≤ −0.3) and identified 25,991 age-related sites (9% of the dataset). Ninety-seven percent of the age-related sites gained DNA methylation with age, while 3% of the age-related sites lost methylation with age. We aligned the 2759 aging sites of the DREAM discovery dataset with the 450K probes of the 25,991 aging sites in the TCGA dataset, and 146 unique sites overlapped at 250 bp distance between *SmaI* sites and 450K probes. We restricted the distance between the CpG sites to 250 bp as it has been previously shown that co-methylation over short distances (≤ 1000 bp) is significantly correlated, and this correlation is lost for distances > 2000 bp [33, 34]. We think this stringent cutoff (250bp) helps in decreasing the background noise and increasing the specificity of identifying age-related sites across two different platforms. Therefore, 146 of the age-related sites in the discovery dataset validated in the TCGA dataset. These 146 aging sites were distributed in the following genomic loci: 8.9% in pCGI, 13.7% in pnCGI, 25.3% in npCGI, and 52.1% in npnCGI regions (Additional file 2: Figure S2 a, b). In comparing these loci to the genomic distribution of all the sites (45,135) in the discovery dataset (Additional file 2: Figure S2 c), aging sites were more likely to be in pnCGI and npCGI (OR 2.4, 95% CI 1.48–3.82 *p* value = 0.0003 and OR 2.52, 95% CI 1.74–3.67 *p* value < 0.0001, respectively). Furthermore, these validated aging sites significantly correlated in their direction of change with age in all the genomic contexts (Additional file 2: Figure S2 d) across the two assay platforms.

### Age-related methylation changes in cancer

**Fig. 2:**
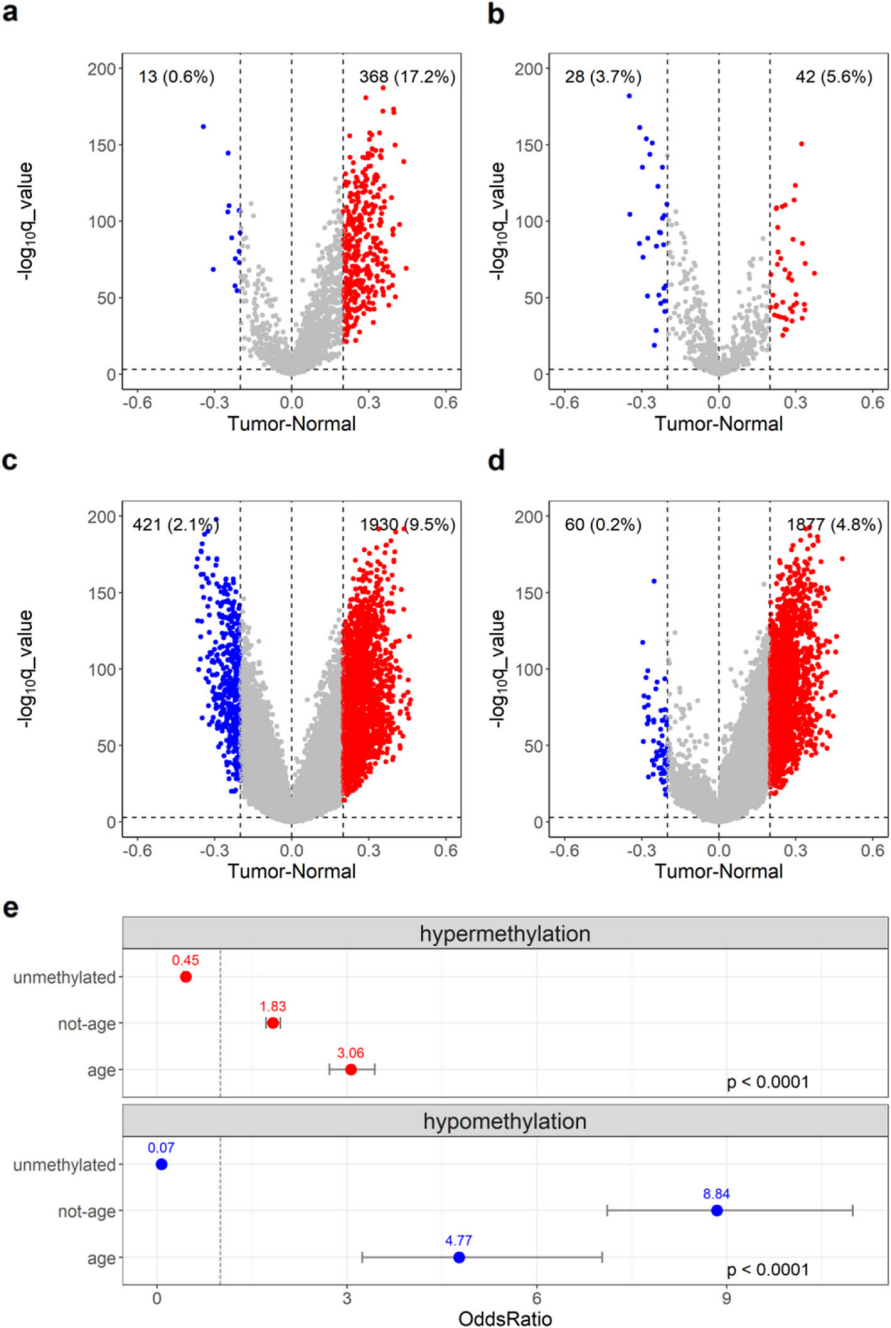
Age-related DNA methylation sites in normal-adjacent breast tissue gain methylation in breast cancer. **a**–**d** Volcano plots of age-related hypermethylated (**a**), age-related hypomethylated (**b**), not age-related (**c**), and unmethylated sites (**d**) in TCGA breast tumors compared to normal samples. The *x*-axis is the methylation difference between the average methylation (*β* values) for 784 breast tumors and 97 normal-adjacent samples; the sites with a methylation difference between tumor and normal of at least 20% are indicated in red (hypermethylation) or in blue (hypomethylation). The *y*-axis is the –log10 of *q* value. Cutoff at the *y*-axis is at –log10 (*q* value) = 3. **e** Summary of odds ratios: the upper panel is for hypermethylation in unmethylated, not-age, and age sites compared to hypermethylated sites in all the datasets, and the lower panel is for hypomethylation with comparisons done in the same categories. The numbers on top of the dots represent the estimates of the odds ratio, and the error bars represent the 95% confidence intervals around the estimates. All comparisons were tested for significance by a chi-square test (*p* value < 0.0001)

We next compared the methylation changes in purified breast epithelia to the methylation levels in TCGA normal adjacent tissue (*n* = 97) and to the TCGA breast cancer tissue (*n* = 784). To be able to compare DREAM data to the 450K array data, we first aligned DREAM (*SmaI*) sites to TCGA normal adjacent 450K probes at an absolute distance of no more than 250 bp. As in previous reports [20], the age-related sites we identified in the purified breast epithelium showed a gain of DNA methylation in TCGA breast tumors (Fig. 2a). The sites that did not show changes in DNA methylation with age (empirical *p* value ≥ 0.05, Spearman rho < 0.3 or > −0.3) were referred to as not age-dependent sites (Fig. 2c). The unmethylated sites in the breast epithelium (less than 1% methylation by DREAM) (Fig. 2d) also gained methylation in TCGA cancer. However, as shown in the upper panel of Fig. 2e, compared to all the other data, age-related sites were the best predictors of hypermethylation in cancer, with an odds ratio of 3.06 and *p* value < 0.0001. On the other hand, there were fewer age-related hypomethylated sites in TCGA cancer (Fig. 2b, blue), and unlike in the case of hypermethylation, age-related hypomethylated sites were not the best predictors of hypomethylation in cancer (Fig. 2e, blue). This could be explained by the 450K array’s bias towards promoter regions and making it less likely to pick up hypomethylation of non-promoter regions.

### DNA methylation datasets

While examining the age-dependent DNA methylation changes in the TCGA normal adjacent dataset, we noted a few samples that were outliers in terms of DNA methylation levels. To systematically look for these outlier samples, we used 450K methylation data for 6 datasets (normal samples from GEO datasets GSE88883, GSE101961, GSE74214, normal-adjacent samples from TCGA, and both normal and normal adjacent samples from GSE69914). The characteristics of the samples are summarized in Table 1. Next, we ran a principal component analysis on the validated 146 age-dependent DNA methylation values in 427 patients. The summary of PCA analysis and the scatter plots for the first 3 components are shown in Additional file 3: Figure S3. The highest proportion of variance was explained by age (PC1), and the data showed a uniform group of patients with no apparent strong batch effect, but several outliers were immediately apparent in the plot.

**Table 1:** Breast tissue DNA methylation datasets in this study

Dataset	Age range	Median age	Mean age	SD age	Sample size	Outlier	Type	Method
GSE88883	18–82	37	37.2	13.6	100	0	N	450K
GSE74214	13–80	54.5	49	20.8	18	3	N	450K
GSE101961	17–76	38	38.2	12.2	121	2	N	450K
GSE69914	18–80	51	49.5	14.4	49	10	N	450K
GSE69914	30–86	51	51.1	12.2	42	7	N-adj	450K
GSE160233	33–82	50	52	14.3	29	NA	N-adj	DREAM
TCGA	28–90	56.5	57.5	15.3	97	17	N-adj	450K

DNA methylation datasets in normal and normal-adjacent breast tissues. Summary of the characteristics of publicly available and in-house generated DNA methylation datasets used in this study. Raw (idat) files for all 450K datasets were downloaded and were re-normalized within themselves to match the normalization of the GSE69914 dataset for which raw files were not available for normalization. The data were normalized using beta-mixture quantile normalization (BMIQ) through the ChAMP R package. DREAM dataset was generated in-house as described in the “Methods” section. The “Outlier” column indicates the number of outliers identified in each dataset

*N* normal, *N-adj* normal-adjacent, *NA* not applicable

### Outliers of DNA methylation are more prevalent in normal-adjacent breast samples

**Fig. 3:**
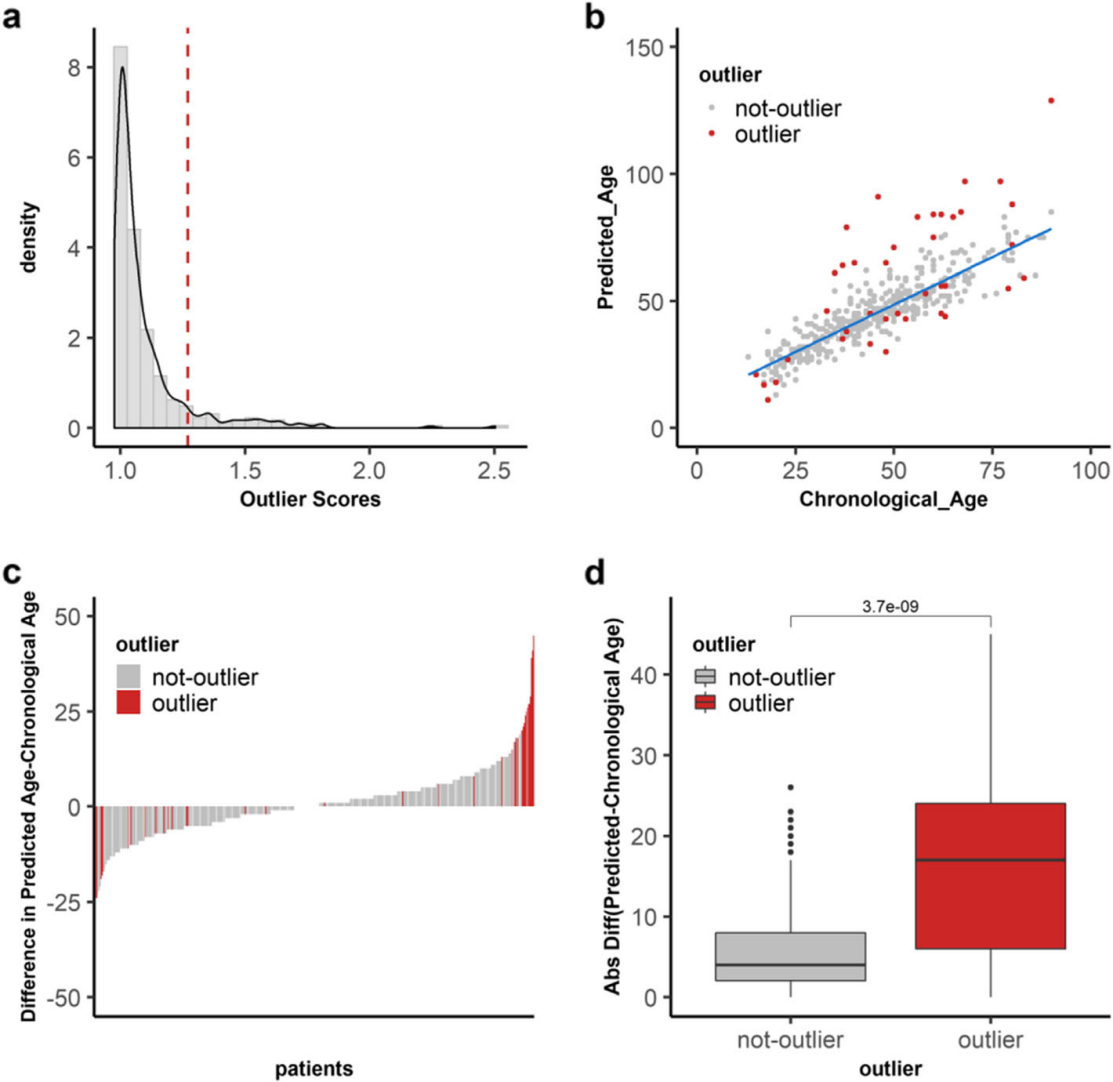
DNA methylation outliers are identified through age-dependent sites. **a** Distribution of the outlier scores (*x*-axis) calculated by the local outlier factor algorithm on 427 patients from six DNA methylation datasets. The dashed line represents the cutoff for outlier designation based on LOF outlier scores of ≥ Q3 + 1.5 × IQR (red line) across all patients. **b** A Lasso regression model was built on the DNA methylation values of 146 aging sites in all non-outlier samples (gray) from 6 datasets to predict the ages of the samples. The chronological ages and the predicted ages for 427 patients are shown in the scatter plot with outlier samples in red. **c** The difference between the predicted age and the chronological age (*y*-axis) is shown in the bar plot for 427 patients (*x*-axis). The 39 outlier patients are in red while the not-outliers are in gray. **d** Comparison of the absolute difference between the predicted age and the chronological age on the *y*-axis for not-outlier (gray) and outlier (red) samples on the *x*-axis. Significance was tested by the Wilcoxon test

**Fig. 4:**
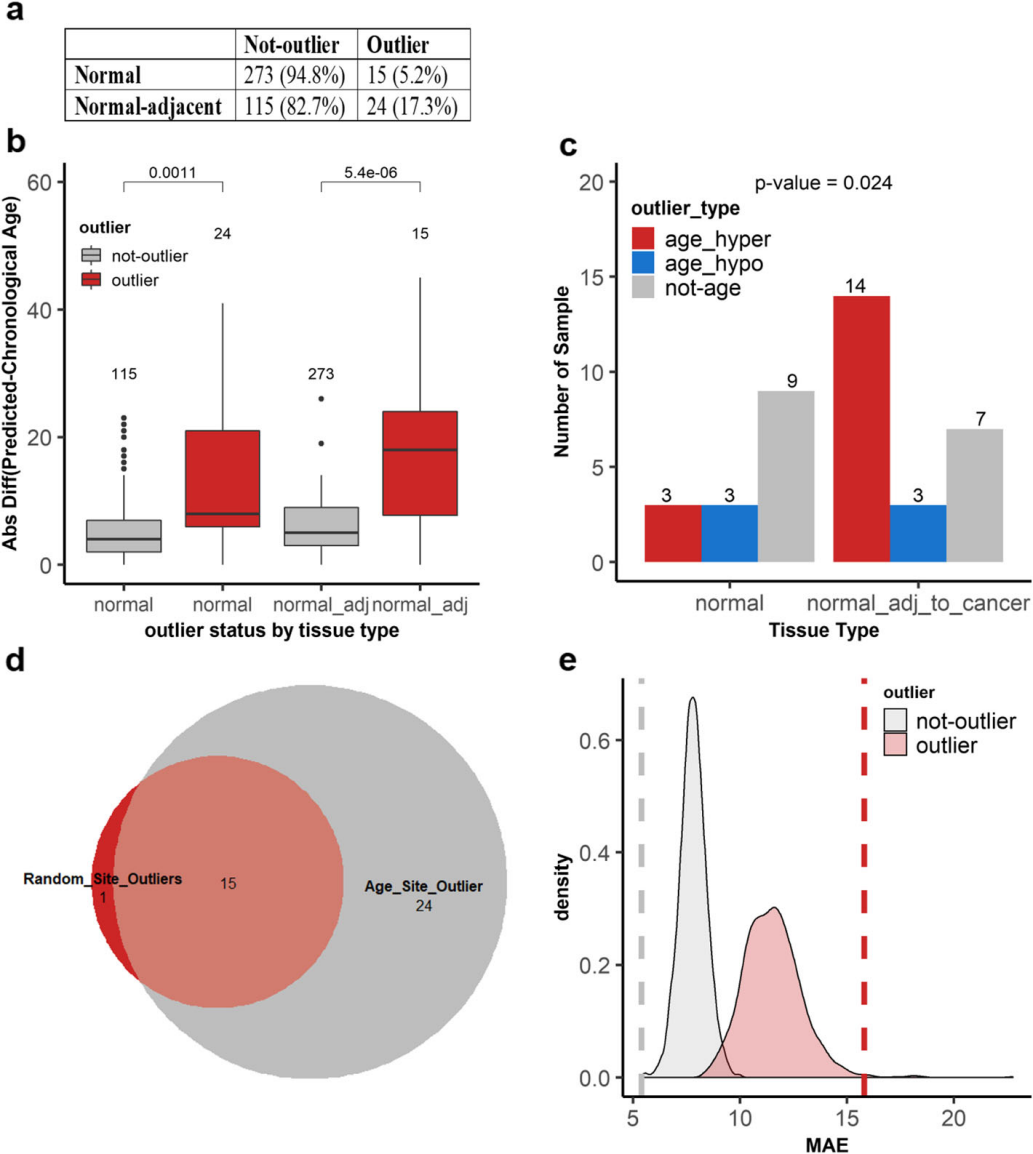
Outlier samples are more prevalent in tumor-adjacent samples and exhibit accelerated aging. **a** A table indicating the number of outliers and not-outliers in normal adjacent and normal samples. The proportion of samples in each group is represented by percentages, and significance was tested by the Fisher exact test (*p* value = 0.0001, OR=3.9, 95% CI 1.8–8.1). **b** The absolute difference between the predicted and the chronological age on the *y*-axis for not-outlier (gray) and outlier (red) samples in normal samples and normal adjacent to cancer samples. The numbers above the box plots represent the number of samples in each group. Significance was calculated by the Wilcoxon test. **c** The outliers are represented in three groups in both normal and normal adjacent to cancer tissues. The numbers on the bar graphs represent the number of samples in each group. In red are outliers with an age difference of increase of 10 years older or more between the predicted age and the chronological age, in blue are outliers with an age difference of 10 years younger or lower, and in gray are outliers with an age difference between 10 years younger and 10 years older. **d** Overlap of outlier patient samples identified by 146 age-related sites and outliers identified by random sites in 95% of the permutations. The significance of overlap was tested by the hypergeometric test (*p* value = 2.23 × 10^−16^). **e** Prediction evaluation of the difference between the chronological age and the predicted age in outliers defined by random sites or by aging sites. The *x*-axis is the mean absolute error (MAE) of the difference between the chronological age and the predicted age for each sample in each of the 1000 randomly generated outlier detection models. The red density plot represents the distribution of MAE of outliers from the 1000 predicted models; the gray density plot is the distribution of the not-outlier from the same 1000 predictions. The red (outlier) and gray (not-outlier) dashed lines represent the MAE values based on the aging sites

To detect the outlier patients, we calculated a local outlier factor score using parameter *k* = 20. We found that 39 out of the 427 (9.1%) patient samples had outlier score values of greater than Q3 + 1.5 × IQR (Fig. 3a). Several groups have reported that age-related methylated sites can be used to predict biological ages. Hence, we reasoned that one plausible difference between these 39 outliers and the remaining 388 not-outlier samples is a difference in their biological ages relative to their chronological ages. Therefore, to predict the biological ages, we built a Lasso model on the methylation values of the 146 aging sites of the not-outlier samples (Fig. 3b). We then applied the model to the outlier samples to predict their biological ages and to compare them to their chronological ages. As shown in Fig. 3c, we noted that the difference in the predicted and the chronological ages for outliers was much greater than that of the not-outlier samples. To measure these differences statistically, we compared the absolute differences between the predicted and the chronological ages (Fig. 3d). Outliers had a median value of 17 while not-outliers a median value of 4. This difference was significant by the Wilcoxon test (*p* value = 3.7 × 10^−9^). Interestingly, we also found that there were significantly more outlier samples in normal-adjacent to cancer (24/139, 17.3%) than in normal samples (15/288, 5.2%) (*χ*^2^ = 16.4, *p* = 0.0005) (Fig. 4a). Additionally, the absolute differences between the predicted and the chronological ages among outlier and not-outliers were significant both in normal (*p* value = 0.0011) as well as in normal-adjacent samples (*p* value = 5.4 × 10^−6^). Significance was measured by the Wilcoxon test (Fig. 4b).

### Outlier samples are enriched for the accelerated aging phenotype in normal adjacent breast samples

We noted the differences in the aging phenotype of the different outlier samples. There were three different outlier types: accelerated aging outliers whose predicted ages were older than their chronological ages by at least 10 years (Fig. 3c, right-hand side), decelerated aging outliers whose predicted ages were younger by at least 10 years (Fig. 3c, left-hand side) ,and then there were those DNA methylation outliers with predicted versus chronological age differences of less than 10 years (Fig. 3c, middle). We found that accelerated aging outliers were enriched at a higher frequency in normal-adjacent to cancer (14/17, 82%) compared to normal samples from patients without cancer (3/17, 18%) (Fig. 4c) (*p* value = 0.024, Fisher’s exact test). To confirm that our selected age-dependent sites are more reliable at detecting outlier patients than random sites, we constructed a null distribution by randomly sampling 146 sites from the 450K data 1000 times, followed by applying the same approach of PCA analysis then LOF to predict the outliers and Lasso to regress their ages. Sixteen samples from the permutation analysis were considered as outliers because they were identified in 95% of the permutations based on standard statistical practices. Fifteen of the 39 outliers detected by age-dependent DNA methylation sites overlapped with the 16 outliers detected by the random sites (Fig. 4d). Though the overlap was significant by the hypergeometric test (*p* value = 2.23 × 10^−16^), age-related sites identified distinct outliers. Next, we calculated the mean absolute error (MAE) between the chronological age and the predicted age of the 1000 random iterations that identified the random outlier samples. The distribution of the MAE values for the random outliers (red) and the random not-outliers (gray) are shown in Fig. 4e. The MAE values for age-dependent outliers and not-outliers are indicated by the dashed lines (red and gray, respectively). Though the outlier patients identified by age-dependent sites or random sites largely overlapped, the MAE values differed. The MAE value for outliers identified by age-dependent sites was higher (15.8, red dashed line) than the distribution of MAE values of the random outliers. This suggests that the outlier status can be detected by many CpG sites throughout the genome, but that age-dependent sites detect distinct outliers and are better at detecting the accelerated aging phenotype in outlier samples. Furthermore, we also investigated how Horvath’s multi-tissue estimator clock performed in detecting the outliers on the DNA methylation datasets in the current study (Additional file 4: Figure S4). To achieve this, first, we checked how many of the 353 Horvath’s CpG are in the TCGA dataset, and second, we investigated how many of those sites are aging sites in our permutation-based age identification model. We found that 347 CpG sites are in the TCGA dataset, but only 32 of those sites (~9%) are aging sites in the same dataset and none of those 32 sites overlapped with the validated 146 aging sites. Despite this, to identify outliers using the 347 CpG probes, we applied our outlier analysis model and found 18 outliers in the TCGA dataset. However, there was an insignificant overlap between the outlier samples identified by Horvath’s sites and by our 146 aging sites (*p* value = 0.06). More importantly, Horvath’s CpG sites could only detect one TCGA age-accelerated sample out of the 10 accelerated outliers identified by our 146 aging sites.

### Outlier status in cancer tissue

**Fig. 5:**
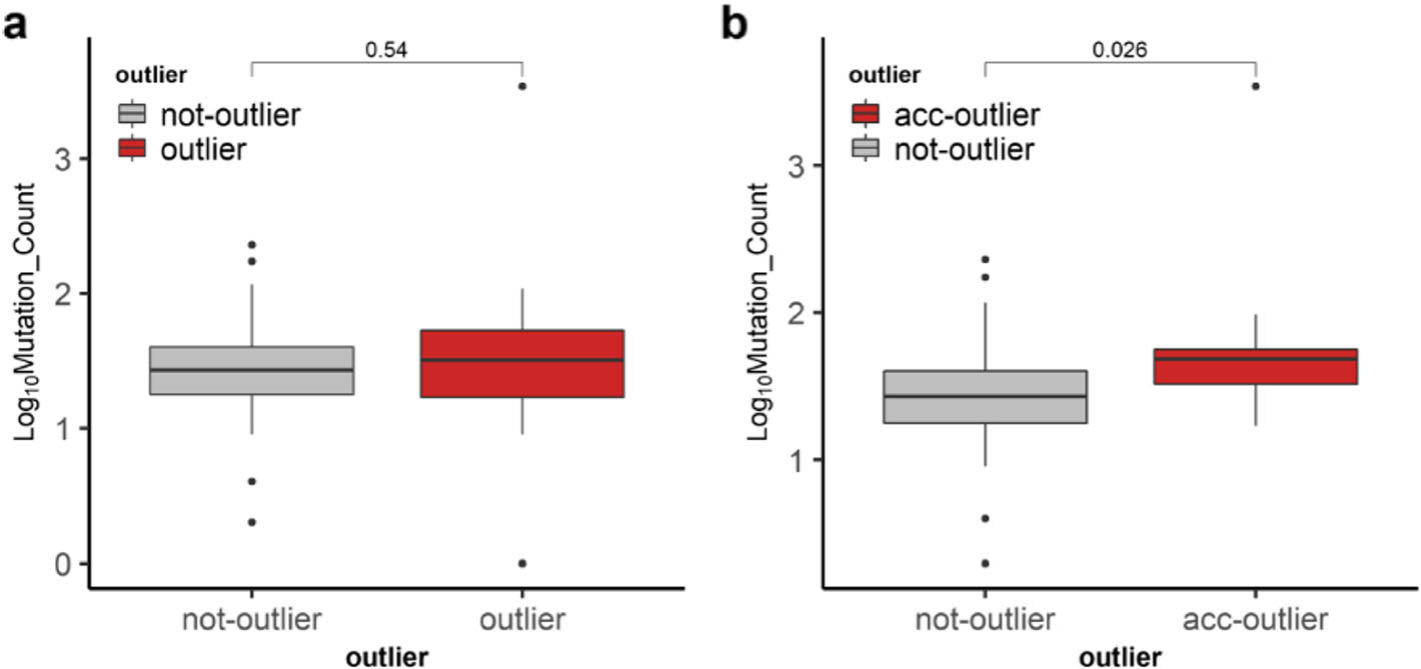
Mutation frequencies in the TCGA samples. Mutation counts were downloaded from the cBioPortal. Comparison of mutation count (*x*-axis is the outlier status; *y*-axis is the log of mutation count) was done between outliers and not-outlier samples (**a**) and between not-outlier and accelerated outliers (acc-outlier) (**b**). Significance was tested by the Wilcoxon test

**Fig. 6:**
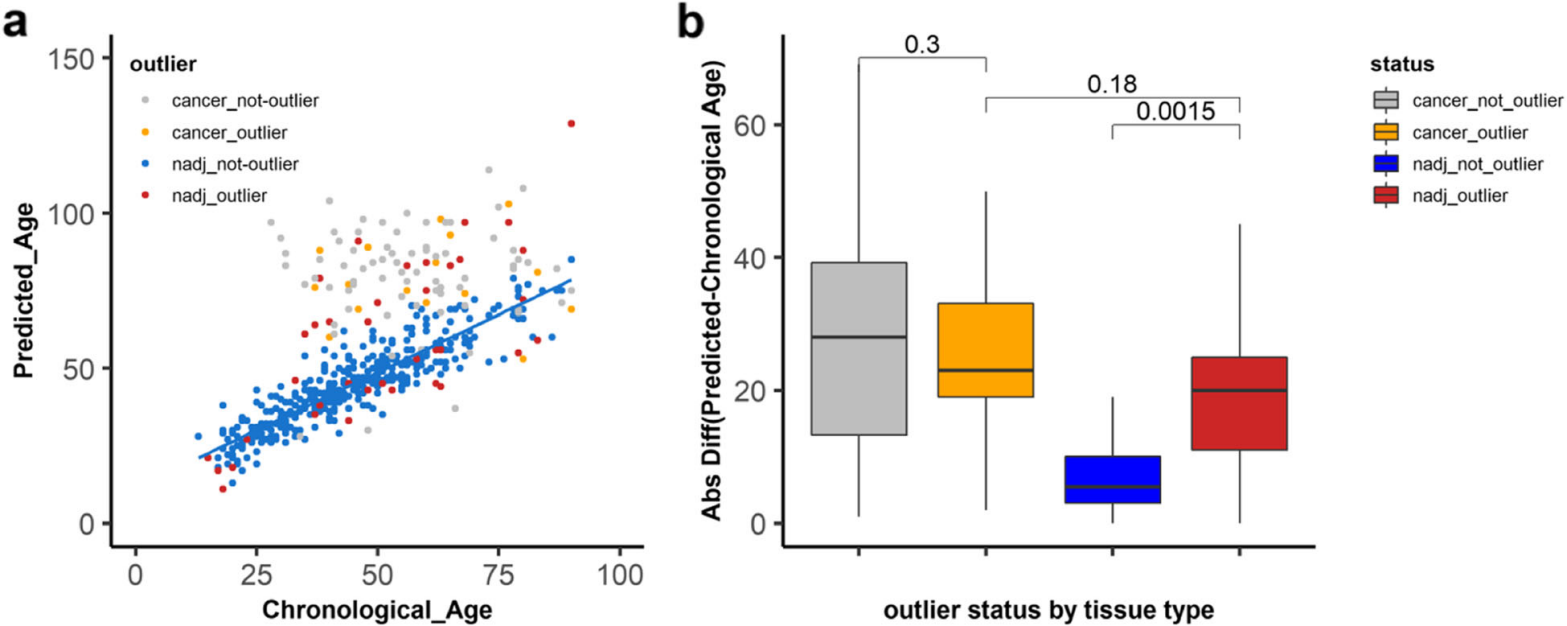
Outlier analysis of TCGA normal adjacent and breast cancer samples. **a** A Lasso regression model was built on the DNA methylation values of 146 aging sites in all non-outlier samples from the six DNA methylation datasets (blue) to predict the ages of all other samples: the outlier samples in normal adjacent (red), the not-outlier cancer samples (gray), and the outlier cancer samples (orange). The chronological ages (*x*-axis) and the predicted ages (*y*-axis) are shown in the scatter plot. **b** Comparison of the absolute difference between the predicted ages and the chronological ages (*y*-axis) in outliers and not-outliers in normal adjacent (red, blue) and in cancer (orange, gray). Significance was tested by the Kruskal-Wallis test followed by a post hoc Dunn test with multiple testing correction using the Benjamini-Hochberg method

Because the TCGA data has annotated clinical data, we next focused on these 97 samples out of the 427 samples. We wanted to find out if the differences between not-outlier, outlier, and accelerated outlier samples can be explained by any of the clinical parameters. We did not find a significant difference for the type of breast cancer, molecular subtype, menopause status, race, prior history of cancer, or stage of the disease (Additional file 5: Table S1). The only significant difference was in the mean predicted age (older in accelerated outliers) (*p* value = 1.62 × 10^−8^). Next, we wanted to find out if there was a potential clinical correlation between DNA methylation outliers and the mutation load. To this end, we used the TCGA Firehose Legacy mutation data, which gives the mutation count for all nonsynonymous mutations, for 97 patients from cBioPortal. The difference in mutation count between outliers and not-outliers was not significant (Fig. 5a), while the difference between accelerated outliers and not-outliers was significant (*p* value = 0.026) by the Wilcoxon test (Fig. 5b). We next asked whether the outlier phenotype is carried forward to cancer and studied the cancer samples corresponding to the normal-adjacent samples (*n* = 91). Comparing the differences between the predicted and chronological ages across the groups, we found that, although cancer samples had larger differences than the normal adjacent samples, the difference between outliers and not-outliers in the TCGA cancer samples was not significant (*p* value = 0.3) (Fig. 6b orange and gray). However, the difference between the outliers and not-outliers in the normal-adjacent samples was significant (*p* value = 0.0015) (Fig. 6b, red and blue). Furthermore, the difference between cancer outliers and normal-adjacent outliers was not statistically significant (*p* value = 0.18) (Fig. 6b, orange and red). Statistical significance was tested by the Kruskal-Wallis test followed by a post hoc Dunn test with multiple testing corrections using the Benjamini-Hochberg method. This suggests that there is a limit to the accelerated aging phenotype, which is reached in cancer, thus minimizing the differences between the cancer outliers and cancer not-outliers. This also indicates that the outliers in the normal adjacent tissue are severely altered since they are not different than the cancer outliers.

## Discussion

In this study, we show age-dependent DNA methylation drifts in normal breast tissue and that these changes, especially the hypermethylated perturbations, are reflected in the breast cancer tissues. We also highlight that outlier DNA methylation patterns are more frequently found in the normal-adjacent tissues of women with cancer and their unique accelerated aging phenotype in the normal adjacent (pre-malignant) tissue.

In previous studies, DNA methylation perturbations due to age in breast tissue were identified and were used to predict biological ages using the Horvath clock (353 CpG) [35–37]. However, in our study, instead of relying on a clock that was devised across multiple tissues and did not differentiate tissue-specific age-related changes (Additional file 4: Figure S4), we identified CpG sites using permutation tests in purified mammary epithelial cells. This approach also eliminated variation in fat content that is reported to vary among normal breast tissue samples [11].

Our findings that age-related hypermethylated and hypomethylated sites are enriched in different genomic regions (promoter CpG islands and non-promoter non-CpG islands, respectively) are consistent with previous reports [20, 21]. However, unlike in previous publications, we identified more sites that hypomethylate with age. This inconsistency could be explained by the different methodologies used to measure the methylation levels. Previous studies used 27K or 450K arrays for methylation which were designed to cover gene promoters and therefore are less likely to pick up hypomethylation of non-promoter regions [38]. Even in our own permutation analysis of the TCGA normal adjacent data (450K array), we only found a handful of sites that hypomethylated with age (Additional file 1: Figure 1) further highlighting the array’s bias. Indeed, using odds ratio calculations, we show that non-promoter non-CGI sites are more likely to be detected in DREAM compared to 450K array while the promoter non-CGI sites were favored in 450K array (Additional file 6: Figure S5). Additionally, we cannot rule out that some of the hypomethylation could have been due to culturing of the purified mammary epithelial cells for 4 passages prior to DNA extraction as it was previously described for other human primary cells [39].

Our findings that age-related hypermethylated sites in a normal breast are enriched in breast cancer are in line with previous studies [20–22, 40]. Additionally, our findings that age-related hypermethylated sites are the best predictors of hypermethylation in cancer highlight the far-reaching implications of using methylation levels of these sites to predict pre-neoplastic changes.

There are several reports that describe how DNA methylation alterations in normal cells that are enriched in cancer cells are predictive of tumorigenesis [11, 12]. These alterations are rare events, and their identification has been challenging. In previous studies, Teschendorff et al. [11, 12] clearly raise the point that to identify rare heterogenous stochastic events, one should use differentially variable and differentially methylated CpG sites because mean methylation differences would miss those rare events. Our current study supports this concept as our unsupervised anomaly detection algorithm does not assume homogeneity and detects outliers by distance dissimilarity within the population. Importantly, the uniqueness of our study includes identification of the outliers using tissue-specific age-related methylation changes (rather than tissue-agnostic clocks), and the statistical approach to identifying outliers, which is also agnostic of whether the outliers show accelerated vs. disordered age. Our findings establish that outlier samples are more frequently present in tissues adjacent to the cancer compared to the normal and are characterized by an accelerated aging (predicted age older than the chronological age) phenotype. These findings indicate that age-related DNA methylation changes could be used to identify these rare outliers and their distinct biological phenotype, which could not be identified by random sites nor by Horvath’s multi-tissue estimator CpG sites (Fig. 4d and Additional file 4: Figure S4). This is an interesting distinction because one important factor affecting outlier samples is epigenetic age, which could present a greater risk of age-related diseases such as cancer. On the other hand, our findings that mutation load is significantly different between accelerated aging outliers and not-outliers (Fig. 5b) could indicate one potential clinical correlation between DNA methylation outliers (epigenetic changes) and mutation frequency (genetic changes). We are aware though that this difference can be attributed to the one sample that has the highest mutation frequency and excluding that sample returned a *p* value of 0.07 (data not shown). However, in the future, with the availability of more samples, this could be further explored. Additionally, there is mounting evidence that chronic inflammation could result in DNA methylation abnormalities that in this context could explain the outlier status. However, this is a possibility that remains to be determined in future studies.

Another interesting finding in our study is that although age-related sites do change DNA methylation from preneoplastic tissue to cancer tissue, all cancer samples had the accelerated aging phenotype, and there was no significant difference between cancer outliers and outliers of the normal adjacent tissues. This contrasted with Teschendorff’s studies, where they showed a progressive change in DNA methylation from normal to preneoplastic tissue and to cancer tissue, and this change was exacerbated in cancer in the outlier samples. In our study, this change is not further extended in cancer tissues of the outliers possibly because in cancer samples, the disrupted epigenome has reached the maximum possible acceleration and cannot accelerate any further based on DNA methylation levels. This is also suggestive of additional numerous DNA methylation abnormalities that define the cancer epigenome irrespective of the outlier status. This also highlights the severity of the outliers of the pre-malignant tissue which is no different than the cancer outliers. Therefore, identifying these outlier individuals based on age-related DNA methylation sites can potentially stratify individuals whose strikingly altered epigenome looks like the alteration observed in cancer. In future studies, it is important to investigate whether these epigenetic changes are present in blood samples or as circulating DNA in cell-free preparations to warrant their potential use as clinical biomarkers for early detection and/or for monitoring levels and possibly reversal of the alterations by lifestyle changes such as calorie restriction.

## Conclusions

The data presented in this study suggests that age-dependent DNA methylation outlier profiles in pre-malignant tissue are infrequent events but have strikingly altered epigenome like in cancer that has far-reaching clinical implications for early detection and possibly intervention by lifestyle changes.

## Supplementary Information

**Additional file 1.** Identification and validation of age-related DNA methylation sites in TCGA breast normal adjacent tissue. a) TCGA 97 normal-adjacent samples were used as validation dataset for the aging sites. One thousand permutations of the data were performed, and empirical p-values were computed. The distribution of the Spearman correlation r values of the actual dataset is shown in pink while the distribution of the correlation values obtained by random permutation analysis of the same data is shown in green. b) The age-dependent methylation changes were selected based on a cutoff of permutation empirical p-value (p < 0.05) and based on Spearman correlation of r ≥ 0.3 (gain of methylation with age) and r ≤ −0.3 (loss of methylation with age). The age-dependent sites from the discovery dataset (DREAM) and the validation dataset (450K array), were aligned and restricted to < 250 bp distance between SmaI sites and the 450K probes.

**Additional file 2.** Genomic distribution of validated aging sites. a) Distribution of the 146 aging sites within the promoter CpG islands (pCGI), promoter non CpG islands (pnCGI), non-promoter CpG islands (npCGI) and non-promoter non CpG islands (npnCGI). b) Distribution of all sites (45,135) within the same genomic context as in (a) in the discovery dataset. c) Summary of odds ratios of the genomic region specificity of age-related sites (146) compared to all sites (45,135) in the discovery dataset. Dots represent the point estimates of odds ratio with lines representing 95% confidence intervals around the estimates. A chi-square test was used to test for statistical significance for each comparison; p-values for all comparisons were significant. d) Contingency tables of the shared 146 aging sites between the DREAM and array platforms. Each table represents a genomic context, and the numbers indicate the number of CpG for hyper (hypermethylated) or hypo (hypomethylated) sites. All comparisons were significant with p-values < 0.0001.

**Additional file 3.** PCA analysis of age-related methylation sites. a) Summary of PCA analysis of the methylation values of 146 age-dependent probes in 6 publicly available datasets. Proportion of variance explained by each of the first 10 principal components (PC). b) Scatter plots of PC components 1 and 2, c) components 2 and 3, and d) components 1 and 3.

**Additional file 4.** Outlier analysis using Horvath’s multi-tissue estimator clock’s 353 CpG probes. a) Flow chart of the identification of age-related sites in TCGA. Bold numbers in parentheses indicate the number of Horvath clock’s CpGs present based on the cut-off. b) Venn diagram showing no overlap between the clock’s 32 CpG sites and the validated 146 aging sites. c) Venn diagram showing the overlap between outlier samples identified by Horvath clock’s CpG sites and the outliers identified by our 146 aging sites. the significance of overlap was tested by the hypergeometric test and found to be insignificant (p= 0.06). d) Overlap of the accelerated outliers identified by the clock’s CpG sites in the TCGA dataset and the accelerated outliers identified by our aging sites in the same dataset.

**Additional file 5.** Comparison of TCGA clinical parameters. Comparison of different variables was done between the not-outlier and outlier samples and accelerated outliers. The outlier status in this analysis was based off the aging methylation data of 427 patients. Chron Mean Age and Pred Mean Age are chronological and predicted average ages respectively. Mixed ductal lobular carcinoma (MDLC), Invasive ductal carcinoma (IDC), Invasive lobular carcinoma (ILC). TN is triple negative. The significant p-value was generated by ANOVA testing followed by Tukey’s HSD post hoc test.

**Additional file 6.** Genomic context specificity of different methylation platforms. Bar plots of the odds ratios (y-axis) of all sites (left) and of all aging sites (right) in DREAM to 450K array. X-axis is the genomic context of all comparisons. All comparisons were tested for significance by a chi-square test and stars indicate p-values < 0.0001.

## Data Availability

The dataset generated and analyzed during the current study is available from the corresponding author on request. In addition, the DNA methylation profiles discussed in this publication have been deposited in NCBI’s Gene Expression Omnibus database under accession number GSE160233.

## Abbreviations

3′ UTR: 3′ untranslated region
5′ UTR: 5′ untranslated region
Acc-outlier: Accelerated outlier
BMIQ: Beta-mixture quantile
CGI: CpG island
CH3: Methyl
CpG/CG: Cytosine followed by guanosine
Chron mean age: Chronological mean age
DREAM: Digital Restriction Enzyme Analysis of Methylation
HMEC: Human mammary epithelial cell
IDC: Invasive ductal carcinoma
ILC: Invasive lobular carcinoma
IQR: Interquartile range
Lasso: Least absolute shrinkage and selection operator
LOF: Local outlier factor
MAE: Mean absolute error
MDLC: Mixed ductal lobular carcinoma
nCGI: Non-CpG island
npCGI: Non-promoter CpG island
npnCGI: Non-promoter non CpG island
OR: Odds ratio
PCA: Principal component analysis
pCGI: Promoter CpG island
pnCGI: Promoter non CpG island
pred mean age: Predicted mean age
TCGA: The Cancer Genome Atlas
TN: Triple negative
TSS: Transcription start site
