# Supplementary material for: Accelerated aging in normal breast tissue of women with breast cancer
Source: Breast Cancer Res. 2021 May 22;23:58. doi: 10.1186/s13058-021-01434-7 (PMC8140515; doi:10.1186/s13058-021-01434-7)

Additional file 1: Figure S1 Identification and validation of age-related DNA methylation sites in TCGA breast normal adjacent tissue.

a

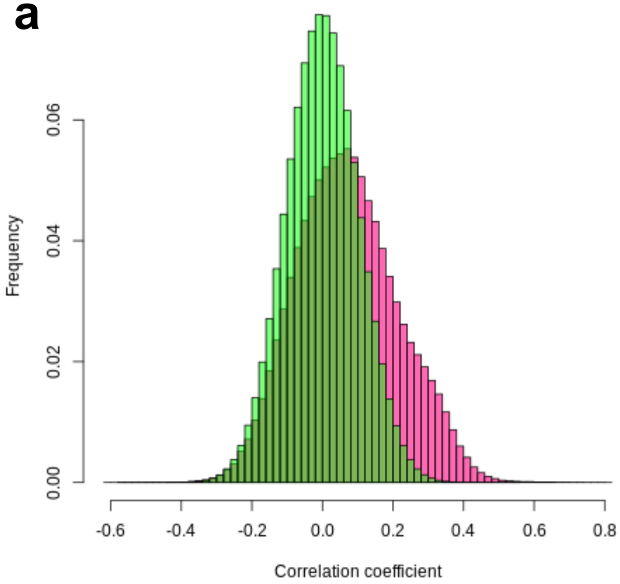

b

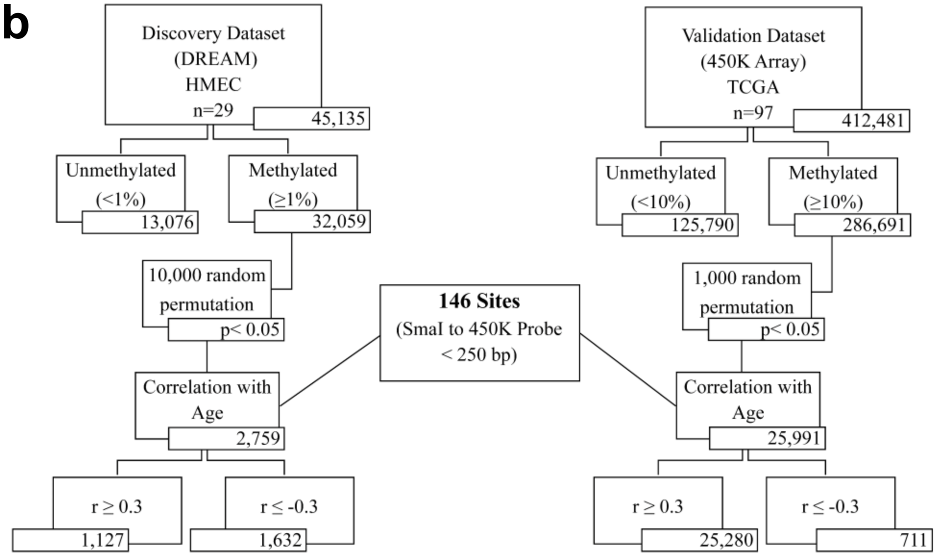

Supplement: Supplementary file 1 — Additional file 1. Identification and validation of age-related DNA methylation sites in TCGA breast normal adjacent tissue. a) TCGA 97 normal-adjacent samples were used as validation dataset for the aging sites. One thousand permutations of the data were performed, and empirical p-values were computed. The distribution of the Spearman correlation r values of the actual dataset is shown in pink while the distribution of the correlation values obtained by random permutation analysis of the same data is shown in green. b) The age-dependent methylation changes were selected based on a cutoff of permutation empirical p-value (p < 0.05) and based on Spearman correlation of r ≥ 0.3 (gain of methylation with age) and r ≤ −0.3 (loss of methylation with age). The age-dependent sites from the discovery dataset (DREAM) and the validation dataset (450K array), were aligned and restricted to < 250 bp distance between SmaI sites and the 450K probes. [file 13058_2021_1434_MOESM1_ESM.pdf]
