# Supplementary material for: Accelerated aging in normal breast tissue of women with breast cancer
Source: Breast Cancer Res. 2021 May 22;23:58. doi: 10.1186/s13058-021-01434-7 (PMC8140515; doi:10.1186/s13058-021-01434-7)

Additional file 2: Figure S2 Genomic distribution of validated aging sites and direction of age-related changes across assay platforms

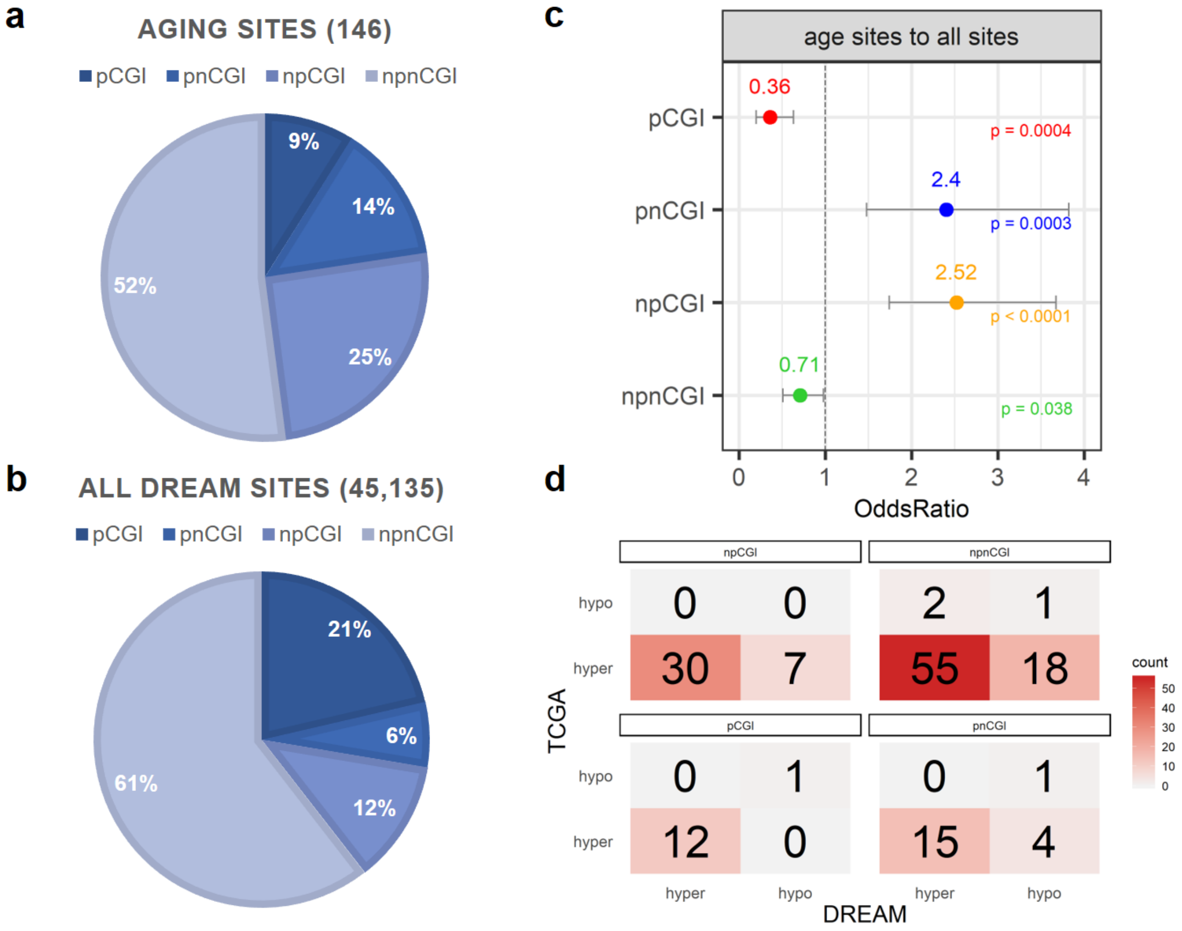

Supplement: Supplementary file 2 — Additional file 2. Genomic distribution of validated aging sites. a) Distribution of the 146 aging sites within the promoter CpG islands (pCGI), promoter non CpG islands (pnCGI), non-promoter CpG islands (npCGI) and non-promoter non CpG islands (npnCGI). b) Distribution of all sites (45,135) within the same genomic context as in (a) in the discovery dataset. c) Summary of odds ratios of the genomic region specificity of age-related sites (146) compared to all sites (45,135) in the discovery dataset. Dots represent the point estimates of odds ratio with lines representing 95% confidence intervals around the estimates. A chi-square test was used to test for statistical significance for each comparison; p-values for all comparisons were significant. d) Contingency tables of the shared 146 aging sites between the DREAM and array platforms. Each table represents a genomic context, and the numbers indicate the number of CpG for hyper (hypermethylated) or hypo (hypomethylated) sites. All comparisons were significant with p-values < 0.0001. [file 13058_2021_1434_MOESM2_ESM.pdf]
