# Supplementary figures and images for: Accelerated aging in normal breast tissue of women with breast cancer
Source: Breast Cancer Res. 2021 May 22;23:58. doi: 10.1186/s13058-021-01434-7 (PMC8140515; doi:10.1186/s13058-021-01434-7)

### Additional file 3: Figure S3 PCA analysis of age-related methylation sites

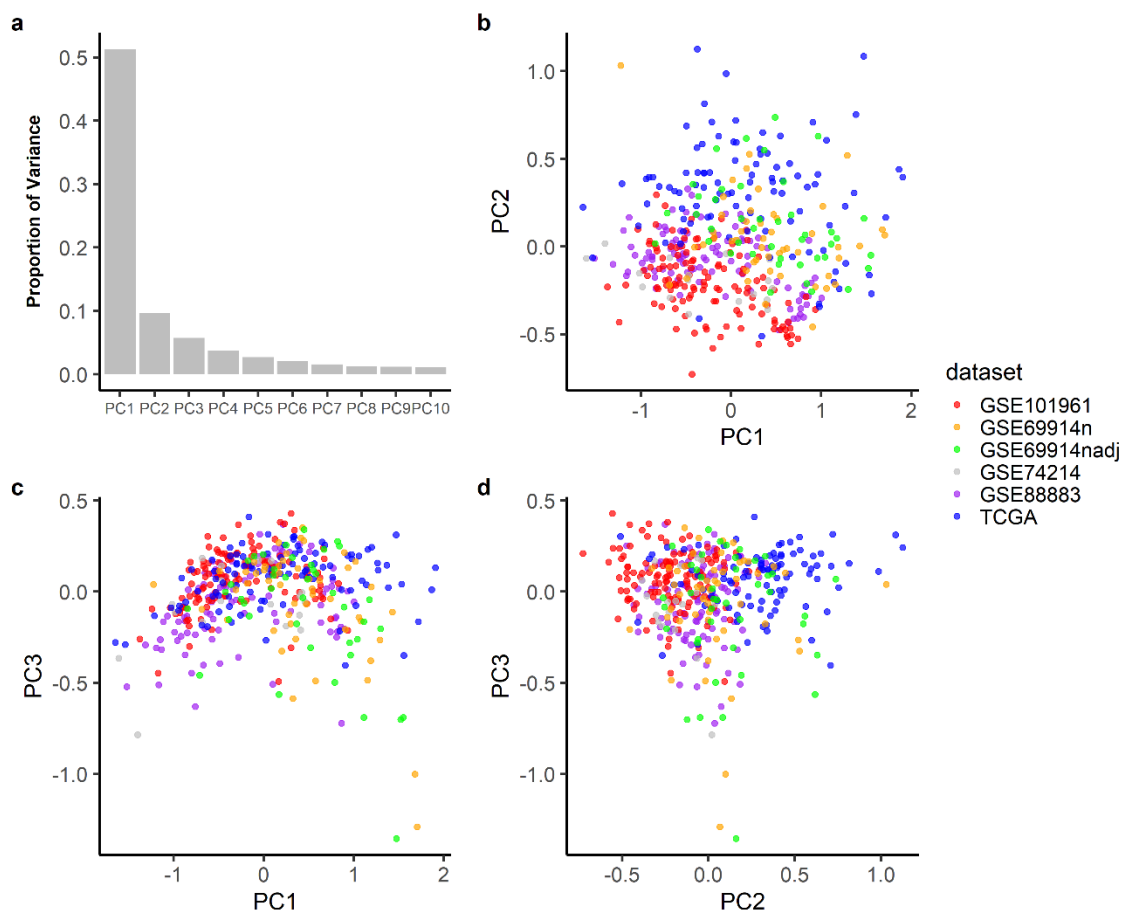

Supplement: Supplementary file 3 — Additional file 3. PCA analysis of age-related methylation sites. a) Summary of PCA analysis of the methylation values of 146 age-dependent probes in 6 publicly available datasets. Proportion of variance explained by each of the first 10 principal components (PC). b) Scatter plots of PC components 1 and 2, c) components 2 and 3, and d) components 1 and 3. [file 13058_2021_1434_MOESM3_ESM.pdf]

Additional file 4: Figure S4 Outlier analysis using Horvath’s 353 CpG probes

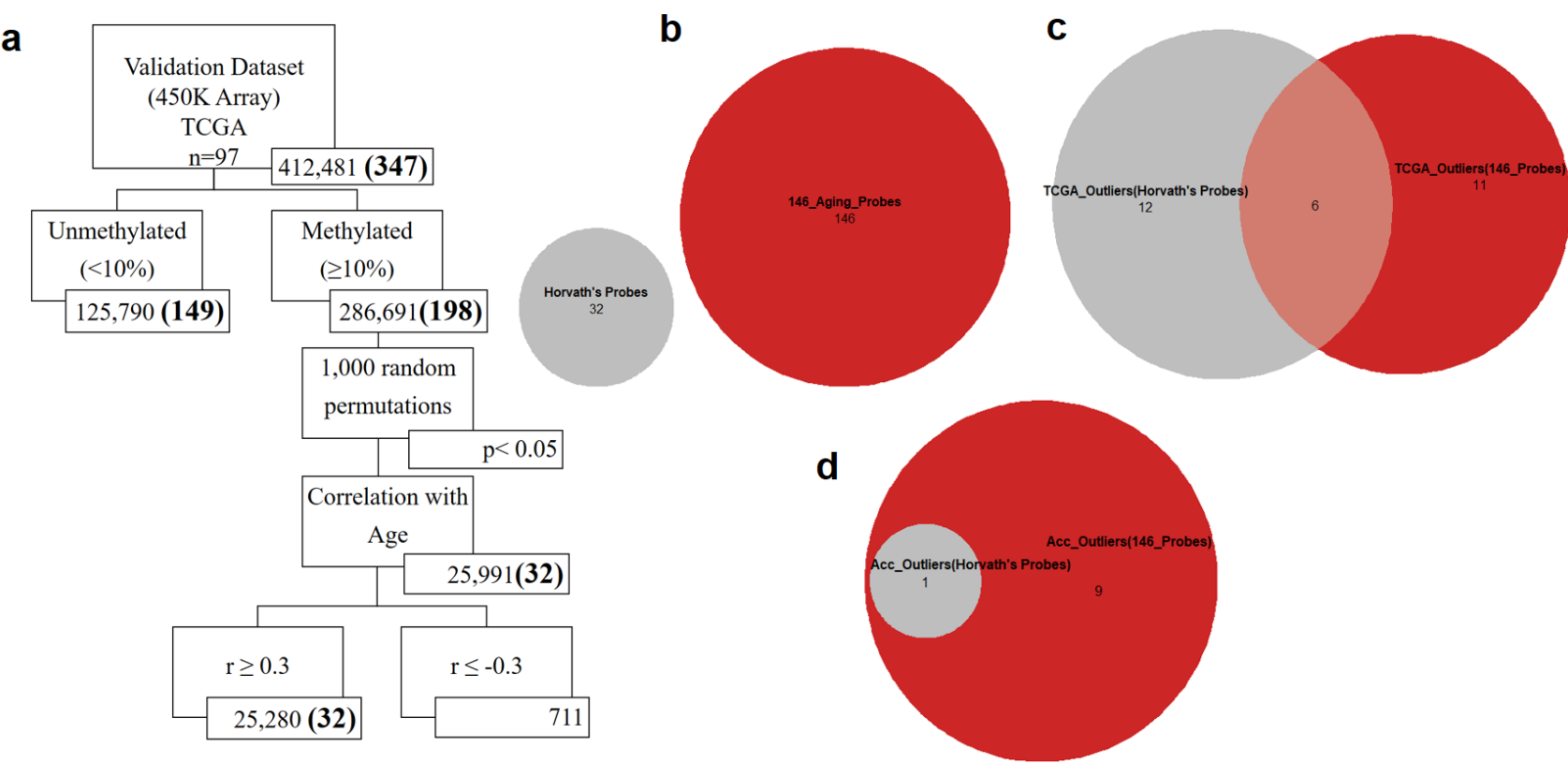

Supplement: Supplementary file 4 — Additional file 4. Outlier analysis using Horvath’s multi-tissue estimator clock’s 353 CpG probes. a) Flow chart of the identification of age-related sites in TCGA. Bold numbers in parentheses indicate the number of Horvath clock’s CpGs present based on the cut-off. b) Venn diagram showing no overlap between the clock’s 32 CpG sites and the validated 146 aging sites. c) Venn diagram showing the overlap between outlier samples identified by Horvath clock’s CpG sites and the outliers identified by our 146 aging sites. the significance of overlap was tested by the hypergeometric test and found to be insignificant (p= 0.06). d) Overlap of the accelerated outliers identified by the clock’s CpG sites in the TCGA dataset and the accelerated outliers identified by our aging sites in the same dataset. [file 13058_2021_1434_MOESM4_ESM.pdf]

**Additional file 6: Figure S5 Genomic context specificity across DREAM and 450K assay platforms**

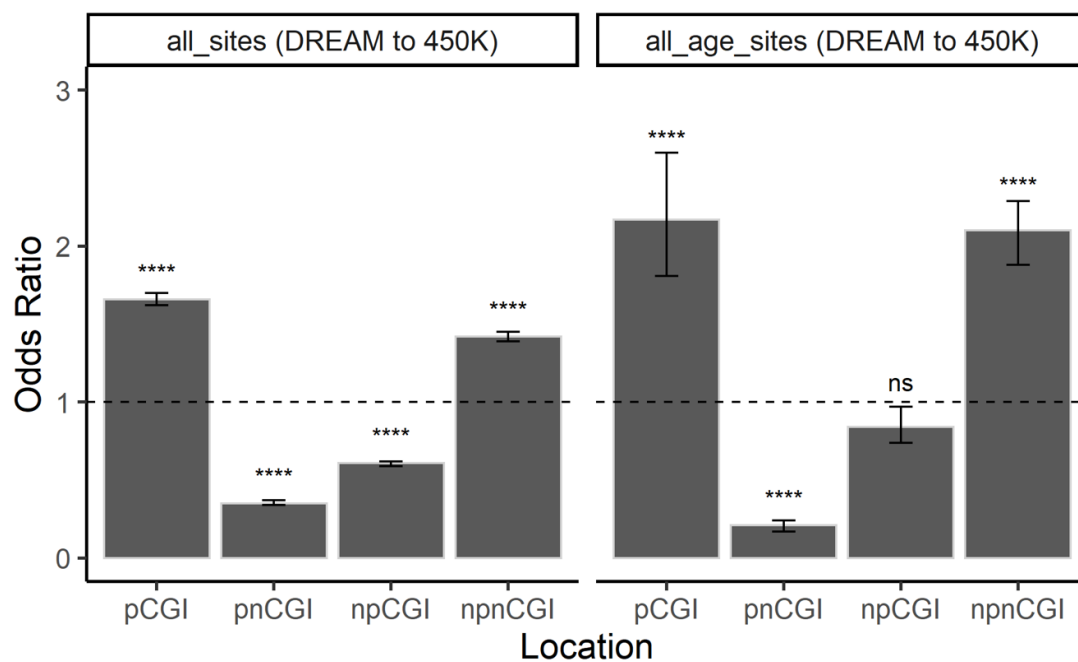

Supplement: Supplementary file 6 — Additional file 6. Genomic context specificity of different methylation platforms. Bar plots of the odds ratios (y-axis) of all sites (left) and of all aging sites (right) in DREAM to 450K array. X-axis is the genomic context of all comparisons. All comparisons were tested for significance by a chi-square test and stars indicate p-values < 0.0001. [file 13058_2021_1434_MOESM6_ESM.pdf]
