# Supplementary material for: Accelerated aging in normal breast tissue of women with breast cancer
Source: Breast Cancer Res. 2021 May 22;23:58. doi: 10.1186/s13058-021-01434-7 (PMC8140515; doi:10.1186/s13058-021-01434-7)
Supplement: Supplementary file 5 — Additional file 5. Comparison of TCGA clinical parameters. Comparison of different variables was done between the not-outlier and outlier samples and accelerated outliers. The outlier status in this analysis was based off the aging methylation data of 427 patients. Chron Mean Age and Pred Mean Age are chronological and predicted average ages respectively. Mixed ductal lobular carcinoma (MDLC), Invasive ductal carcinoma (IDC), Invasive lobular carcinoma (ILC). TN is triple negative. The significant p-value was generated by ANOVA testing followed by Tukey’s HSD post hoc test. [file 13058_2021_1434_MOESM5_ESM.pdf]

Additional file 5: Table S1 clinical parameters of the TCGA dataset

	Not Outlier (n=80)	Outlier (not accelerated) (n=7)	Accelerated Outlier (n=10)	p-value
Chron Mean Age	57	58	61	ns
**Pred Mean Age**	**56**	**50**	**88**	**$1.62x10^{-8}$**
Stage I	9/79 (11%)	3/7 (43%)	2/10 (20%)	ns
Stage II A, B	52/79 (66%)	4/7 (57%)	5/10 (50%)	ns
Stage III A, B, C	17/79 (22%)	0/7 (57%)	3/10 (30%)	ns
Stage IV	1/79 (1.2%)	0/7	0/10	ns
MDLC	8/80 (10%)	0/7	1/10 (10%)	ns
IDC	64/80(80%)	7/7 (100%)	8/10 (80%)	ns
ILC	7/80 (9%)	0/7	1/10 (10%)	ns
Paget's disease	1/80 (1.3%)	0/7	0/8	ns
Post Menopause	44/66 (67%)	4/7 (57%)	6/8(75%)	ns
Pre-menopause	20/66 (30%)	3/7 (43%)	2/8 (25%)	ns
Perimenopause	2/66 (3%)	0/7	0/8	ns
Asian	1/79 (1.2%)	0/7	0/7	ns
Black or African American	3/79 (3.8%)	0/7	1/10 (10%)	ns
White	75/79 (95%)	7/7 (100%)	9/10 (90%)	ns
Prior cancer	2/80 (2.5%)	0/7	1/10 (10%)	ns
ER+	59/72 (82%)	5/7 (71%)	5/6 (83%)	ns
HER2+	3/61 (4.9%)	0/6	0/5	Ns
TN	7/61 (11%)	1/6 (17%)	1/5(20%)	ns

Comparison of TCGA clinical parameters. Comparison of different variables was done between the not-outlier and outlier samples and accelerated outliers. The outlier status in this analysis was based off the aging methylation data of 427 patients. Chron Mean Age and Pred Mean Age are chronological and predicted average ages respectively. Mixed ductal lobular carcinoma (MDLC), Invasive ductal carcinoma (IDC), Invasive lobular carcinoma (ILC). TN is triple negative. The significant p-value was generated by ANOVA testing followed by Tukey's HSD post hoc test.
